# The Advancement of In Vitro Lipolysis: Two-Step Flow-Through Method for the Evaluation of Lipid-Based Drug Delivery Systems

**DOI:** 10.3390/pharmaceutics17050545

**Published:** 2025-04-23

**Authors:** Katarina Rede, Mirjana Gašperlin, Marija Bogataj, Katarina Bolko Seljak

**Affiliations:** Faculty of Pharmacy, University of Ljubljana, Aškerčeva cesta 7, 1000 Ljubljana, Sloveniamirjana.gasperlin@ffa.uni-lj.si (M.G.); marija.bogataj@ffa.uni-lj.si (M.B.)

**Keywords:** in vitro lipolysis, flow-through dissolution system, drug release, self-microemulsifying drug delivery systems

## Abstract

**Objectives**: A novel two-step flow-through in vitro lipolysis model was developed for the evaluation of drug release from a self-microemulsifying drug delivery system (SMEDDS). **Methods**: Firstly, the SMEDDS was dispersed in an acidic medium. Subsequently, the pH was increased, and a lipolytic reaction was immediately initiated, accompanied by medium flow onset. The latter enabled increase of the initial low pH of the medium, improving the physiological relevance of the method by simulating dosage form retainment in the stomach and transfer to the duodenum, which is very important for a weakly basic active pharmaceutical ingredient (API) incorporated in an SMEDDS. **Results**: Conversely to the traditional pH-stat in vitro lipolysis, the developed method is not established on titration, as the reaction vessel pH is regulated by a medium flow and buffer capacity. Individual parameters, such as pancreatin activity, buffer capacity, and medium shift, were researched using traditional pH-stat in vitro lipolysis prior to their implementation in the flow-through setup. **Conclusions**: The concentration of the solubilized model API, carvedilol, was increased as pancreatin activity decreased and as buffer capacity increased. The ratios between release profiles obtained under different conditions utilizing the pH-stat and novel two-step flow-through in vitro lipolysis were comparable; however, the differences were more pronounced in the flow-through method.

## 1. Introduction

Orally administered medicines are exposed to digestion enzymes in the gastrointestinal tract, which can alter the composition of some drug delivery systems. For lipid-based drug delivery systems (LBDDS), commonly formulated to improve poor solubility of APIs, lipolytic degradation is particularly important because the formulation’s solubilization capacity can be changed because of enzymatic activity, and hence its drug release and absorption can change. To evaluate drug release from LBDDS, in vitro digestion methods have been developed [1,2,3].

pH-stat in vitro lipolysis is the most commonly used in vitro digestion method for the evaluation of LBDDS, particularly self-microemulsifying drug delivery systems (SMEDDSs). It is based on a neutralization titration, which maintains the pH in the reaction vessel constant during the lipolytic reaction. Lipid excipients are digested by lipases, and as a product of the lipolysis, free fatty acids are formed, which lower the pH in the reaction vessel but are then neutralized. The enzyme source added is usually porcine pancreatin; therefore, only the intestinal phase of lipolytic degradation is simulated. The digestion process is evaluated by the volume of the added titrant, which is usually a NaOH solution, and by the concentration of the solubilized and precipitated drug in the samples that are withdrawn during the reaction [1,4].

Following the oral administration of LBDDSs, many complex processes, such as dispersion, lipolytic activity, the formation of colloidal structures, the inclusion of endogenous surfactants, etc., impact the formulation and the behavior of the drug [2,3]. Traditional pH-stat in vitro lipolysis primarily simulates enzymatic degradation, yet it often lacks in vivo predictability. Therefore, novel in vitro digestion models and optimizations of the pH-stat in vitro lipolysis model are being developed with a common objective of improving the biorelevance of the in vitro digestion model and thus correlation with in vivo data [1].

One of the potential ways to ensure improved predictability of in vitro lipolysis is the incorporation of the gastric step, as in vivo LBDDSs are first dispersed in the gastric fluid; moreover, lipid digestion starts in the stomach. Furthermore, gastric emptying controls the transfer of the dispersion to the duodenum [1,2,5,6]. LBDDSs can be dispersed in an acidic medium [7,8], or, to mimic physiological conditions more closely, acid-stable lipase can be added to initiate the lipolytic reaction already in the acidic environment. The acid-stable lipases used were either recombinant dog gastric lipase (rDGL) or microbial lipase [9,10,11,12,13].

Another concept for improving the physiological relevance of in vitro lipolysis is combining a digestion model with a permeation model [1,2]. Some digestion–permeation models have been used to simultaneously evaluate lipolysis and permeation [14,15], but the majority of them utilize permeation testing after the completion of in vitro lipolysis [16,17,18,19,20].

Undertaking a gastric step or permeation model will transform in vitro lipolysis into a two-step method, hence increasing the number of processes that are simulated. However, some digestion models, such as the INFOGEST model, the TNO gastrointestinal model (TIM), engineered stomach and small intestine (ESIN), and simulator of the gastrointestinal tract (SIMGI), feature multiple processes, parameters, and steps that are present or occur in the gastrointestinal tract. Nevertheless, evaluations using these models are complex and lengthy. Additionally, most of them were originally developed to assess food digestion or to characterize the post-prandial state [21,22,23,24,25,26,27].

Contrary to multi-compartmental and multi-step models, high-throughput in vitro lipolysis was designed for a fast evaluation of LBDDSs. Here, the pH in the reaction vessel is not regulated by titration as in pH-stat in vitro lipolysis but rather by using a medium with a higher buffer capacity [28,29].

Since a number of in vivo evaluations are performed on rats, a rat in vitro lipolysis model was constructed to improve the in vitro–in vivo correlation (IVIVC) for LBDDS. The model is adjusted to the rat gastrointestinal tract by lowering the enzymatic activity and altering the surfactant concentration in the medium. Additionally, medium flow was incorporated in the rat in vitro lipolysis model [7,8]. Moreover, a similar procedure of transferring the digestion content from a gastric to an intestinal compartment was used for the development of in vitro lipolysis simulating the physiology of neonates and young infants [10]. In both dynamic models described, pH in the reaction vessel was regulated via titration using NaOH [7,8,10]. Nevertheless, in vitro lipolysis utilizing medium flow has not yet been applied to a method simulating lipid digestion in adults.

The aim of this study was to develop a biorelevant method featuring lipolysis and exposure of a delivery system to gastric and duodenal environments for the evaluation of drug release from SMEDDSs. Carvedilol was used as a model poorly water-soluble drug. A novel in vitro dissolution method was therefore developed in which in vitro lipolysis was combined with a flow-through dissolution system. Conventional pH-stat in vitro lipolysis served as the basis, which was gradually extended using several biorelevant parameters to improve the ability to evaluate drug release. First, the effects of pancreatin activity and buffering capacity of the medium on the course of in vitro lipolysis were investigated using the traditional pH-stat setup. Then, an additional step was introduced to pH-stat in vitro lipolysis that mimics the dispersion of SMEDDS in acidic gastric fluid and its exposure to low pH values. Furthermore, these improvements were transferred to a flow-through dissolution system, and a two-step flow-through in vitro lipolysis method was developed using medium flow and buffer capacity for pH regulation instead of traditional titration.

## 2. Materials and Methods

### 2.1. Materials

Carvedilol was purchased from Acros Organics, Geel, Belgium. The authors gratefully acknowledge the kind donations of Capmul MCM and Capmul PG-8 by Abitec, Colimbus, OH, USA; Labrasol ALF by Gattefossé, Saint Priest, France; and Kolliphor RH 40 by BASF, Ludwigshafen, Germany. Propylene glycol was purchased from Fagron, Capelle aan den Ijssel, The Netherlands. Trizma^®^ maleate, sodium deoxycholate (NaDC), L-α-phosphatidylcholine (PC), pancreatin from porcine pancreas (≥3 × USP), and 4-bromophenylboronic acid (BBBA) were obtained from Sigma-Aldrich, Burlington, MA, USA. Sodium chloride (NaCl), calcium chloride dihydrate (CaCl_2_ × 2H_2_O), acetic acid (glacial), and potassium dihydrogen phosphate (KH_2_PO_4_) were purchased from Merck, Darmstadt, Germany. Tributyrin was obtained from Carl Roth, Karlsruhe, Germany, and FaSSIF/FeSSIF/FaSSGF powder was obtained from Biorelevant, London, UK. Acetonitrile and methanol were purchased from Honeywell, Charlotte, NC, USA; they were of high-performance liquid chromatography (HPLC) grade. Sodium hydroxide (NaOH) and hydrochloride acid (HCl) concentrates were obtained from Fluka, Buchs, Switzerland. Concentrates were used for preparation of 1 M stock solutions of NaOH and HCl, respectively. Deionized water was used for all the experiments performed. For aqueous mobile phase, ultrapure water was obtained by purification through Mili-Q A10 Advantage purification system (Merck, Darmstadt, Germany).

### 2.2. SMEDDS Preparation

A previously developed SMEDDS with the composition of 15% Capmul MCM, 35% Capmul PG-8, 15% Labrasol ALF, 25% Kolliphor RH 40, and 10% propylene glycol (% are *w*/*w*) was used for the study [30]. All excipients were weighed and thoroughly mixed at room temperature using a magnetic stirrer (Tehtnica, Železniki, Slovenia). Carvedilol powder was added to the SMEDDS at a level of 2% (*w*/*w*) and magnetically stirred for 4 h at room temperature (Tehtnica, Železniki, Slovenia). The entire amount of incorporated carvedilol was dissolved in the SMEDDS. The carvedilol content in the SMEDDS was checked daily.

### 2.3. Preparation of the Media

Simulated gastric fluid (SGFsp) and simulated intestinal fluid (SIFsp) were prepared according to the European Pharmacopoeia [31], but the enzymes were omitted from the media.

Tris-maleate buffers of 2, 25, 50, 100, and 200 mM were prepared by dissolving the appropriate amount of tris-maleate salt, 150 mM NaCl, and 5 mM CaCl_2_ × 2H_2_O in deionized water. A 1 M NaOH solution was added to adjust the pH to 7.5. The concentrated Tris-maleate buffer consisted of the same ingredients as the tris-maleate buffer, but the concentrations of all components used were doubled. The pH of the concentrated buffer was adjusted to 7.8 with 1 M NaOH solution.

The digestion media were prepared by adding NaDC and PC to the Tris-maleate buffers, resulting in a concentration of 4.89 mM and 1.22 mM in the media, respectively. Concentrated digestion medium was prepared as digestion medium, but concentrated tris-maleate buffer was used, and the NaDC and PC concentrations were doubled. Digestion medium and concentrated digestion medium were prepared daily.

Simulated fasting gastric fluid (FaSSGF) was prepared by dissolving 2 g of NaCl in 1 l of 0.01 M HCl solution. Then, 0.06 g FaSSIF/FeSSIF/FaSSGF powder was added to the solution and stirred until it dissolved. The prepared FaSSGF had a pH of around 2. The medium was used up within 48 h.

For the determination of pancreatin activity, a medium mixture of 0.01 M HCl solution and concentrated tris-maleate buffer pH 7.8 was prepared in a ratio of 1:1. The prepared medium mixture had a pH value of 7.5.

### 2.4. Conventional Dissolution Studies

Dissolution studies were performed using an Agilent 708-DS paddle apparatus (Agilent Technologies, Santa Clara, CA, USA). A total of 900 mL of SGFsp or SIFsp was poured into dissolution vessels and heated to 37 °C. Approximately 500 mg of SMEDDS containing carvedilol was added to the dissolution vessels using a syringe. The exact amount of SMEDDS was determined by weighing the difference between the full and empty syringes. During the experiment, the rotation speed of the paddle was set to 75 rpm. Samples were automatically collected at 5, 10, 15, 20, and 30 min (volume of 3 mL) and filtered through 10 µm full flow filters (Agilent Technologies, USA). The collected samples were diluted with methanol, and then the carvedilol concentration was quantified by HPLC. The dissolution experiments were performed in triplicate.

### 2.5. Dynamic Light Scattering Analysis

Dynamic light scattering analysis was performed immediately after completion of the dissolution experiments with samples taken directly from the dissolution vessels. Approximately 1 mL of the sample was transferred to a 12 mm square polystyrene cuvette (Malvern Panalytical, Worcestershire, UK). Droplet size and polydispersity index (PDI) were measured at 25 °C using the Zetasizer Ultra (Malvern Panalytical, Worcestershire, UK).

### 2.6. Determination of Pancreatin Powder Lipolytic Activity

Prior to the in vitro lipolysis experiments, the lipolytic activity of the pancreatin powder was determined using a titrimetric assay procedure, as previously described [32], although some updates were made to the procedure. To prepare the pancreatin stock solution, approximately 50 mg of pancreatin powder was weighed into a test tube, and 10 mL of the selected tris-maleate buffer or medium mixture was added. The prepared dispersion was shaken thoroughly and then centrifuged for 15 min at 5 °C and 4000 rpm in a Centric 400R centrifuge (Tehtnica, Železniki, Slovenia). The prepared stock solution was stored on ice and used within the day.

A pH-stat method using a T70 titrator (Mettler Toledo, Columbus, OH, USA) with a DGi111-SC pH electrode (Mettler Toledo, Columbus, OH, USA) was used to determine the activity of the pancreatin powder. The pH electrode was calibrated daily at 25 °C, and LabX titration pro software (Mettler Toledo, Columbus, OH, USA) was used to monitor the performance of the pH-stat method. A total of 20 mL of the selected tris-maleate buffer or medium mixture was added to a thermostatted vessel and heated to 37 °C. Then, 3 mL of tributyrin was added to the medium, and the pH was automatically adjusted to 7.5 by titration with 0.6 M NaOH. Subsequently, 500 µL of pancreatin stock solution was added to the vessel to initiate the lipolytic reaction. During the 15-min test, the contents of the reaction vessel were continuously stirred and automatically titrated with 0.6 M NaOH solution to maintain the pH at 7.5. The volumetric addition of NaOH was monitored throughout the experiment and then used to calculate the activity of the pancreatin powder. The activity of the pancreatin powder was expressed in TBU/g units. The activity determination was performed in triplicate for each buffer/medium mixture.

### 2.7. Pancreatin Solution Preparation

The pancreatin solution for the in vitro lipolysis experiments was prepared by weighing the pancreatin powder and adding 6 mL of selected tris-maleate buffer. The solution was vortexed thoroughly and centrifuged for 15 min at 5 °C and 4000 rpm in a Centric 400R centrifuge (Tehtnica, Železniki, Slovenia). The mass of the pancreatin powder was calculated based on the previously determined pancreatin powder activity (Section 2.6), and the pancreatin activity was selected for the in vitro lipolysis experiment. The prepared pancreatin solution was stored on ice and used within the day.

### 2.8. pH-Stat In Vitro Lipolysis

The pH-stat in vitro lipolysis experiments were performed using the automated titration equipment and software described in Section 2.6. First, 35 mL of the selected digestion medium with a pH value of 7.5 was added to the thermostated vessel. The medium was heated to 37 °C, then approximately 500 mg of SMEDDS with incorporated carvedilol was added, and the pH was automatically adjusted to 7.5. The lipolysis reaction was initiated by adding 5 mL of the previously prepared pancreatin solution, as described in Section 2.7. The contents of the reaction vessel were continuously stirred during the 30-min test and automatically titrated with 0.6 M NaOH solution to adjust the pH to 7.5. The volumetric addition of NaOH was monitored to estimate the degradation of the lipid excipient. Samples of 1000 µL and 50 µL were taken at 0 (before addition of pancreatin solution), 5, 10, 20, and 30 min. They were manipulated as described below. All pH-stat in vitro lipolysis experiments were performed in triplicate. The average values and SDs were calculated from the results obtained.

#### 2.8.1. Pancreatin Activity Effect

pH-stat in vitro lipolysis was performed with different pancreatin activities—250, 600, and 1000 TBU/mL. In order to achieve the selected pancreatin activity, the mass of the pancreatin powder was adjusted accordingly. The digestion medium used had a tris-maleate concentration of 50 mM.

#### 2.8.2. Buffer Capacity Effect

In addition, pH-stat in vitro lipolysis was performed in the digestion media with different concentrations of tris-maleate buffer. The digestion media used had tris-maleate concentrations of 2, 50, or 200 mM, which also contributed to different buffer capacities of these media. The pancreatin activity was adjusted to 250 TBU/mL.

#### 2.8.3. Two-Step In Vitro Lipolysis

The in vitro lipolysis procedure described above is a one-step pH-stat in vitro lipolysis. A two-stage pH-stat in vitro lipolysis was also developed. For the latter, 20 mL of FaSSGF pH 2 was added to the thermostated vessel. The medium was heated to 37 °C, and then about 500 mg of SMEDDS with incorporated carvedilol was added. The reaction mixture was stirred for 10 min, then samples of 1000 µL and 50 µL were taken. Subsequently, 15 mL of the concentrated digestion medium pH 7.8 was added to the reaction vessel, and the pH was automatically adjusted to 7.5. The rest of the procedure is the same as for the one-step pH-stat in vitro lipolysis method (see above). The tris-maleate concentration after the media change was 50 mM, and the pancreatin activity was adjusted to 250 TBU/mL.

### 2.9. Flow-Through In Vitro Lipolysis

**Figure 1:**
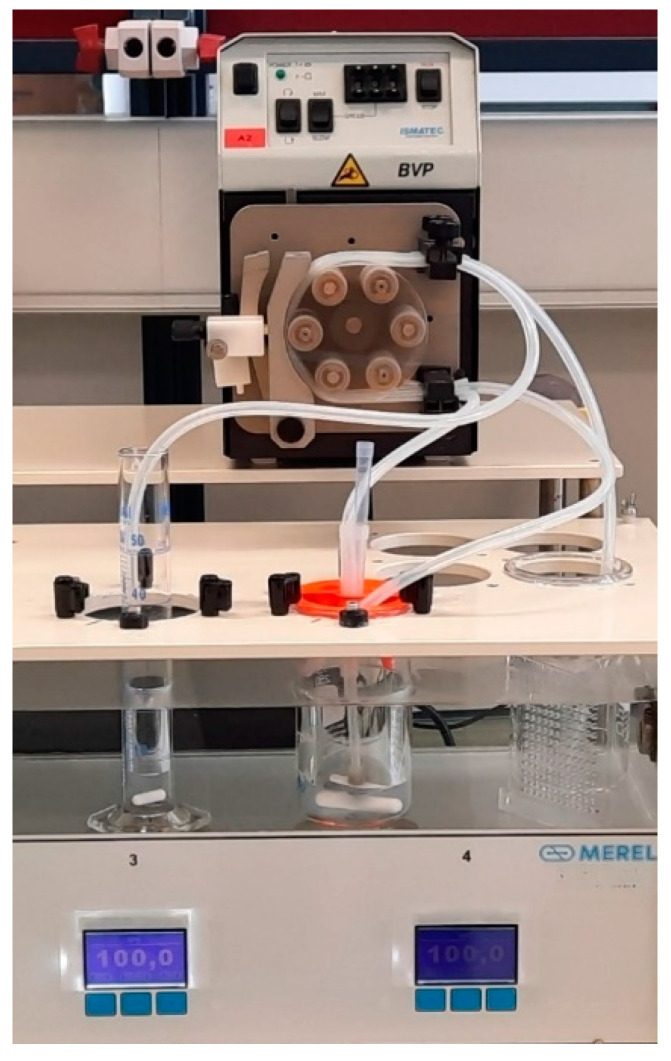
Photo of the flow-through in vitro lipolysis system.

In vitro flow-through lipolysis was performed using a flow-through dissolution system, which has been described in detail previously [33,34]. The flow-through dissolution system was developed at the Faculty of Pharmacy, University of Ljubljana, and built in collaboration with Merel d.o.o., Slovenia [35]. It consists of a thermostatically controlled water bath equipped with vessel holders above magnetic stirrers (Figure 1). A 150 mL glass beaker is used as a reaction vessel, from which samples are pumped out, and fresh medium is pumped continuously using an ISM444B peristaltic pump (Ismatec, Glattburg, Switzerland). The volume of the medium in the reaction vessel is stable due to the constant flow rates. The samples pumped out of the reaction vessel are collected in a measuring cylinder.

For this study, the total volume of medium in the reaction vessel was 40 mL, the magnetic stirrer was set to 100 rpm, the flow rate was 2 mL/min, and a pH electrode was also attached to measure the pH of the reaction vessel contents. As described for the in vitro lipolysis method with pH-stat, 35 mL of the selected digestion medium with a pH value of 7.5 was added to the reaction vessel. The medium was heated to 37 °C, and then about 500 mg of SMEDDS with incorporated carvedilol was added. The lipolysis reaction was initiated by adding 5 mL of the previously prepared pancreatin solution (see Section 2.7). At the same time, the peristaltic pump was switched on to start the flow of medium. The same fresh digestion medium with a pH of 7.5, as in the reaction vessel, was pumped into the reaction vessel, and the samples were pumped out and collected in a measuring cylinder. They were emptied after 5, 10, 15, 20, 30, 40, 50, and 60 min. Immediately afterward, 50 µL and 1000 µL aliquots were withdrawn from the measuring cylinders and manipulated as described below. Throughout the reaction, the pH in the reaction vessel was continuously measured using a FiveEasy Plus™ pH meter (Mettler Toledo, Columbus, OH, USA) with InLab^®^ Micro pH electrode (Mettler Toledo, Columbus, OH, USA). All in vitro flow-through lipolysis experiments were performed in triplicate. The average values and SDs were calculated from the results obtained.

In vitro flow-through lipolysis was performed in the digestion media with different concentrations of tris-maleate buffer, as was the case for in vitro lipolysis with pH-stat. The digestion media used had tris-maleate concentrations of 2, 50, or 200 mM, which also contributed to different buffer capacities of these media. The pancreatin activity was 250 TBU/mL.

In addition, an experiment was performed that fulfilled all the procedural steps described above, but instead of pancreatin solution, only tris-maleate buffer was added to the reaction vessel. The digestion medium used had a tris-maleate concentration of 50 mM.

#### Two-Step In Vitro Lipolysis

The in vitro lipolysis procedure described above is a one-step flow-through in vitro lipolysis. A two-stage flow-through in vitro lipolysis was also developed. The same principles were applied as in the two-step pH-stat in vitro method described in Section 2.8.3: the medium at pH 2 was added to the vessel, SMEDDS was added and stirred for 10 min, the pH of the medium was increased by adding concentrated digestion medium, and pacreatine solution was added to initiate lipolysis. In addition, the peristaltic pump was switched on immediately after the addition of the pancreatin solution. The rest of the procedure is the same as for the one-step flow-through in vitro lipolysis method (described above). The tris-maleate concentration after the medium shift and in the fresh digestion medium pumped into the reaction vessel was 50 mM. The pancreatin activity was 250 TBU/mL.

### 2.10. In Vitro Lipolysis Sample Manipulation

Lipolysis was inhibited in 1000 µL sample by addition of 5 µL of BBBA (1 M solution in methanol). Then, 50 µL samples for the determination of the total carvedilol concentration were diluted to 1000 µL with ice-cold methanol. Both samples were immediately vortexed. The samples were then centrifuged for 10 min at 10 °C and 21,300× *g* in a Centrifuge 5415 R (Eppendorf, Hamburg, Germany). The supernatant of the 50 µL sample was analyzed using HPLC. The 1000 µL samples were separated into two phases during centrifugation—the aqueous phase and the pellet phase. The aqueous phase was diluted 20-fold with methanol and centrifuged again. The supernatant of the diluted samples was analysed using HPLC. The remaining aqueous phase was removed, the pellet phase was dried with a vacuum dryer (Kambič, Semič, Slovenia), and then 1000 µL of methanol was added to the dry pellet phase. The samples were thoroughly mixed for 5 min using a Bullet Blender Homogenizer (Next Advance, USA) at speed 4 and then shaken for one hour at 1200 rpm and room temperature using a HeatMix shaker (Tehtnica, Železniki, Slovenia). They were then centrifuged for 10 min at 10 °C and 21,300× *g* using a Centrifuge 5415 R (Eppendorf, Hamburg, Germany), and the supernatant was analyzed using HPLC. If necessary, the supernatant was additionally diluted with methanol before analysis.

### 2.11. Solid-State Characterization

The pellet phase obtained via one-step and two-step flow-through in vitro lipolysis (50 mM tris-maleate and 250 TBU/mL) was analyzed using X-ray powder diffraction (XRPD) after drying to characterize the solid state of carvedilol. Multiple samples from time points of 5–30 min were combined for analysis. Diffraction patterns were recorded using a powder diffractometer X’PERT PRO MPD PANalytical (Malvern Panalytical, Worcestershire, UK); X-ray beam Cu Kα (λ = 1.542 Å), measurement range: 2–40° 2Θ, excitation voltage: 45 kV, anode current: 40 mA, step size: 0.01° 2Θ, remaining at a step for 0.05 s. The measurement was carried out on a flat sample with a surface/thickness ratio of 10/0.5 mm. The 0.02 rad Soller slits, 10 mm mask, and 1/4° fixed anti-scattering slits were used to correct the primary beam. The irradiated area of the sample was 10 mm; programmable divergent slits were used. The 0.02 rad Soller slits and 5.0 mm anti-scattering slits were used to correct the secondary beam. HighScore Plus software was used to process the diffraction patterns.

### 2.12. Buffer Capacity Determination

Buffer capacities of the tris-maleate buffers and digestion media at pH 7.5 with tris-maleate concentrations of 2, 25, 50, 100, and 200 mM were determined via titration. A 0.1 M solution of HCl or NaOH was added to 40 mL of the medium to pH drop/increase of 0.3 pH unit. pH of the media was measured using SevenCompact™ pH meter (Mettler Toledo, Columbus, OH, USA) equipped with InLab^®^ Expert Pro pH electrode (Mettler Toledo, Columbus, OH, USA). Measurements were performed in triplicate.

### 2.13. Carvedilol Solubility Studies

The solubility of carvedilol was determined in 2, 50, and 200 mM tris-maleate digestion media with a pH of 7.5. About 3 mg of carvedilol was weighed in 1.5 mL micro-centrifuge vials (Eppendorf, Germany), then 1 mL of the medium and about 150 mg of 0.5 mm glass beads were added. The prepared samples were thoroughly mixed for 5 min using a Bullet Blender Homogenizer (Next Advance, Troy, NY, USA) at speed 4. Samples were then shaken in a HeatMix shaker (Tehtnica, Železniki, Slovenia) at 37 °C and 1200 rpm, and after 48 h, they were centrifuged at 37 °C and 21,300× *g* for 10 min. A supernatant aliquot was immediately diluted 20-fold with methanol and analyzed using HPLC. The pH of the remaining supernatant was measured using the SevenEasy™ pH meter (Mettler Toledo, Columbus, OH, USA) equipped with an InLab^®^ Micro pH electrode (Mettler Toledo, Columbus, OH, USA). The study was conducted in four parallel experiments. The average values and SDs were calculated from the results obtained.

### 2.14. HPLC Analysis

Quantitative analysis of carvedilol in samples obtained via in vitro lipolysis, dissolution, and solubility studies was performed using a 1200 series HPLC system (Agilent Technologies, Santa Clara, CA, USA). The basic parameters of the HPLC method were the same as previously [30]. However, the method was scarcely adjusted for carvedilol. A Kinetex C18 (2.10 × 50 mm, 2.6 µm) column (Phenomenex, Torrance, CA, USA) was used with a 0.1% solution of acetic acid and acetonitrile as the mobile phase. The column was heated to 37 °C, the flow rate was set to 0.650 mL/min, and the injection volume was 10 μL. The gradient elution program was as follows: time (min)/% of acetonitrile: 0/20, 2/60, 2.5/60, 2.6/20. Chromatographic separation was achieved in 6 min, with carvedilol retention time being 2.9 min. Quantification was performed using UV detector at 284 nm.

## 3. Results

A novel flow-through dissolution method for SMEDDS evaluation was developed as an upgrade to the traditional pH-stat in vitro lipolysis. The impact of pancreatin activity, medium buffer capacity, and shift of medium pH on drug solubilization during in vitro lipolysis were explored using traditional pH-stat in vitro lipolysis. Employed adaptations were later on gradually applied to the flow-through method, developing an in vitro tool with improved physiological relevance.

### 3.1. Conventional Dissolution Test

**Figure 2:**
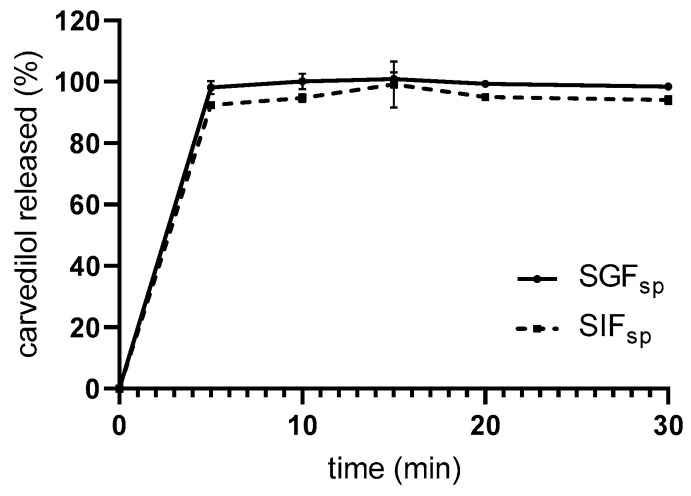
Dissolution profile (mean ± SD; n = 3) of carvedilol from SMEDDS in SGFsp and SIFsp in conventional paddle apparatus.

The dissolution test was performed in a paddle apparatus with SGFsp and SIFsp. After 30 min of dissolution test, 98.5 ± 0.9% and 94.1 ± 1.4% of carvedilol was released in SGFsp and SIFsp, respectively (Figure 2). SMEDDS was able to retain carvedilol in the dissolved form as all of the drug was released immediately, and no precipitation was observed during the experiment; this is especially true for SIFsp as carvedilol is a weak base with pH-dependent solubility [36,37,38]. While no carvedilol precipitation was observed in conventional dissolution, the slightly lower release in SIFsp compared to SGFsp may indicate a minimal extent of carvedilol precipitation. Moreover, in vivo lipid excipient degradation may take place, further facilitating drug precipitation [3,39].

After the dissolution experiment, the size of the dispersion droplets was determined by dynamic light scattering. The droplet diameters in SGFsp and SIFsp were 23.81 ± 0.38 nm and 29.16 ± 4.27 nm, respectively, indicating that the dispersions formed were microemulsions. In both media, the droplet size was similar, and the dispersions were homogeneous (PDI < 0.2).

### 3.2. One-Step pH-Stat In Vitro Lipolysis

#### 3.2.1. Pancreatin Activity Effect

**Figure 3:**
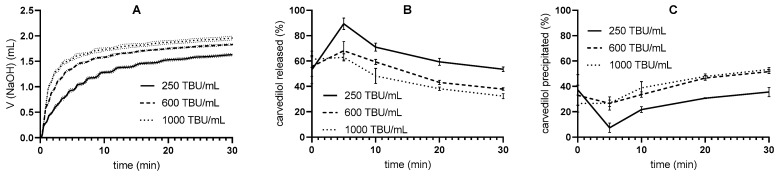
One-step pH-stat in vitro lipolysis results (mean ± SD; n = 3): (**A**) volume of added NaOH, (**B**) carvedilol concentration in aqueous phase, and (**C**) carvedilol concentration in pellet phase for pancreatin levels 250, 600, and 1000 TBU/mL using the 50 mM medium.

The release of carvedilol from SMEDDS was evaluated using pH-stat in vitro lipolysis with three different pancreatin activities—250, 600, and 1000 TBU/mL. During the tests, the volume of titrated NaOH was measured, and the carvedilol concentrations in the aqueous and pellet phases were determined (Figure 3). The drug is dissolved in the aqueous phase and thus represents the portion of the drug that would be absorbed if it were in vivo, whereas in the pellet phase, the drug is in a solid state, meaning that it would likely not be absorbed [1,2,4]. After the initial increase, the concentration of solubilized carvedilol decreased throughout the duration of lipolysis, signifying the loss of the solubilization capacity of SMEDDS caused by the degradation of excipients. Conversely, the percentage of precipitated carvedilol increased with time.

In addition, as pancreatin activity increased, the concentration of solubilized carvedilol decreased, and the concentration of precipitated drug increased, although the difference between precipitated carvedilol at 600 and 1000 TBU/mL was small. The effect of different pancreatin activities on the course of in vitro lipolysis can already be seen in the volume of NaOH used in the titration. After 30 min of reaction time, the mean NaOH volumes were 1.63 ± 0.03 mL, 1.83 ± 0.01 mL, and 1.96 ± 0.04 mL at 250, 600, and 1000 TBU/mL, respectively. The volume of NaOH added increased faster when pancreatin activity was higher.

#### 3.2.2. Buffer Capacity Effect

**Figure 4:**
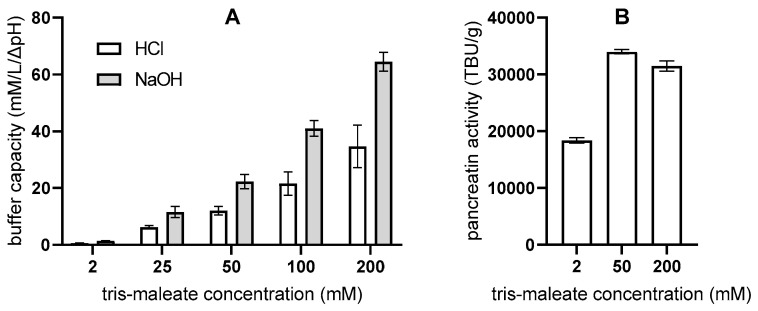
Buffer capacity of media with different tris-maleate buffer concentrations (**A**) and pancreatin activity at three tris-maleate buffer concentrations (**B**); (mean ± SD; n = 3).

The in vitro lipolysis methods described in the literature generally use media with 2 and 50 mM tris-maleate buffers [17,32,40,41,42,43,44], although 25, 100 [45,46], and 200 mM [28,47] tris-maleate buffers are also mentioned. Therefore, the buffering capacities of tris maleate buffers of different concentrations were investigated (Figure 4). Overall, the buffer capacities were higher at higher concentrations of tris maleate. Nearly identical measurements of buffer capacities and the relationships between them were also obtained for the digestion media, i.e., tris-maleate buffer with added NaDC and PC. Nevertheless, differences in buffer capacities can be observed when NaOH or HCl is added, with an increasing trend in both cases (Figure 4).

In addition, the tris maleate concentration had an influence on the measured pancreatin activity (Figure 4). The activity was highest in the 50 mM medium and lowest in the 2 mM medium—no correlation with the buffer capacities was found. Based on the measured pancreatin activities, the mass of the pancreatin powder was adjusted for the in vitro lipolysis experiments.

**Figure 5:**
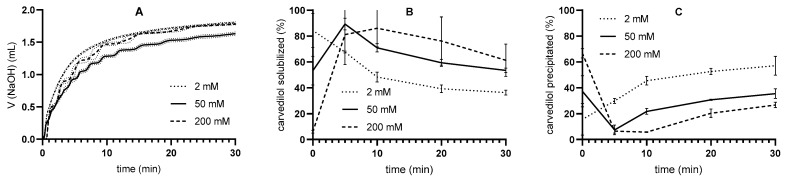
pH-stat in vitro lipolysis results (mean ± SD; n = 3): (**A**) volume of added NaOH, (**B**) carvedilol concentration in aqueous phase, and (**C**) carvedilol concentration in pellet phase for media with 2, 50, and 200 mM of tris-maleate. Pancreatin activity was 250 TBU/mL for all experiments.

In addition to the experiments in 50 mM medium, in vitro lipolysis with pH-stat was also performed in 2 mM and 200 mM media using pancreatin activity of 250 TBU/mL (Figure 5). The pancreatin concentrations used were calculated in accordance with the pancreatin activity/concentration results shown in Figure 4. Interestingly, the volume of NaOH added was the same in both 2 mM and 200 mM media (approximately 1.8 mL after 30 min of reaction time) but slightly lower in the 50 mM medium (approximately 1.6 mL after 30 min of reaction time), suggesting that the degradation of the excipient is not linearly dependent on the buffer capacity of the medium. Nevertheless, the concentrations of solubilized and precipitated carvedilol differed considerably when comparing the release profiles in media with different tris-maleate concentration/buffer capacities. The concentration of solubilized drug after 30 min of lipolysis initiation was the greatest in the 200 mM medium and the lowest in the 2 mM medium. The results for the precipitated drug were reversed.

To possibly explain the effects of tris-maleate concentration/buffer capacity on the release of carvedilol from SMEDDS, the solubility of carvedilol was determined in the 2, 50, and 200 mM digestion media with a pH of 7.5, and the pH of the solutions was then measured after the experiment (Table 1). Carvedilol exhibited similar solubilities in all media tested. The value was highest in the 2 mM medium and lowest in the 200 mM medium, but the differences were small. The pH of the digestion medium before the addition of carvedilol was 7.49 ± 0.01 and did not change during the solubility study, as shown in Table 1. Thus, since there are only minor differences in solubility at different tris-maleate buffer concentrations, this may contribute to, but not be the sole source of, the differences in the pH-stat in vitro lipolysis results shown in Figure 5.

**Figure 6:**
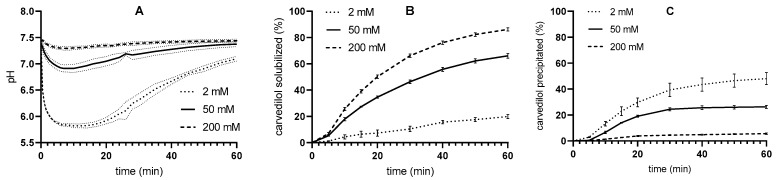
One-step flow-through in vitro lipolysis results (mean ± SD; n = 3): (**A**) pH changes, (**B**) carvedilol concentration in aqueous phase, and (**C**) carvedilol concentration in pellet phase for media with 2, 50, and 200 mM tris-maleate buffer. Pancreatin activity was 250 TBU/mL for all the experiments.

The flow dissolution system developed at the Faculty of Pharmacy of the University of Ljubljana [33] was coupled with in vitro lipolysis. Essentially, SMEDDS was dispersed in 35 mL of medium, and after adding 5 mL of pancreatin solution, the medium flow was switched on. Fresh medium was continuously pumped into the reaction vessel, and samples were fluxed out of it at the same flow rate. Media with different tris-maleate concentrations were used to perform in vitro lipolysis in flow-through. Since the same media were already used for the pH-stat in vitro lipolysis experiments, their buffer capacities were already evaluated. Not surprisingly, at higher buffer capacities, the pH changes during lipolysis were less pronounced than at lower buffer capacities, with the largest pH drop being 1.7 pH units in the 2 mM medium. In the 50 mM and 200 mM media, the pH decreased by a maximum of 0.6 and 0.2 pH units, respectively. Approximately after 10 min of the reaction, the pH started to increase again due to media flow, and after 60 min, the pH in the reaction vessel returned to the initial pH, 7.5, in the 50 mM or 200 mM media. The concentration of solubilized drug was highest when 200 mM medium was used and lowest when 2 mM medium was used. Accordingly, the concentrations of precipitated carvedilol were lowest in 200 mM medium and highest in 2 mM medium (Figure 6).

### 3.3. Two-Step pH-Stat In Vitro Lipolysis

For the in vitro lipolysis model with pH-stat, a two-step procedure was established in which SMEDDS with carvedilol was first dispersed in FaSSGF with a pH of 2, then the pH was increased to 7.5 by adding the concentrated digestion medium, and the lipolytic reaction was initiated by adding pancreatin. The established procedure was later applied to the flow-through dissolution system, resulting in a two-step flow-through in vitro lipolysis method.

**Figure 7:**
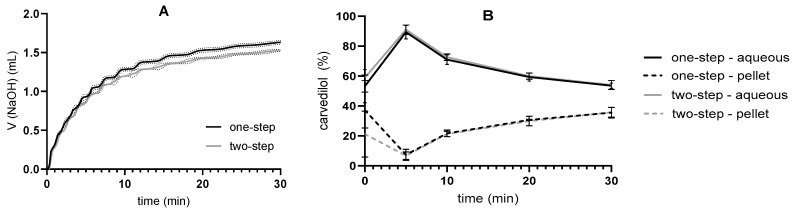
pH-stat in vitro lipolysis results of undertaking one-step and two-step (previous exposure to FaSSGF) procedures (mean ± SD; n = 3): (**A**) volume of added NaOH and (**B**) % of carvedilol in aqueous and pellet phase. Tris-maleate concentration was 50 mM, and pancreatin activity was 250 TBU/mL for both experiments.

Comparing the results of the conventional one-step in vitro lipolysis with the developed two-step method, a smaller volume of NaOH solution was added in the two-step method (Figure 7). However, as the differences were small, the extent of lipolysis without and with prior exposure to an acidic medium is comparable. In addition, the concentrations of dissolved and precipitated carvedilol were the same in both procedures. In FaSSGF, all carvedilol was dissolved, which is due to the ionization of the drug and solubilization by SMEDDS and/or its components. Carvedilol is a weak base with a pKa value of 8.0 and a calculated log P value of 4.0 [38]. Five minutes after exposure to the intestinal medium, partial and progressive precipitation was observed, but the extent of precipitation was comparable to the experiment without prior exposure to the acidic medium.

### 3.4. Two-Step Flow-Through In Vitro Lipolysis

**Figure 8:**
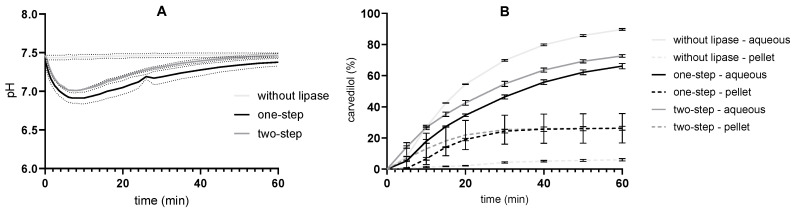
Flow-through in vitro lipolysis results undertaken in one-step and two-step procedures, and without lipase (mean ± SD; n = 3): (**A**) pH changes and (**B**) carvedilol in aqueous and pellet phase. Tris-maleate concentration was 50 mM, and pancreatin activity was 250 TBU/mL for all the experiments.

The two-stage pH-stat procedure described above was used as the basis for two-stage flow-through lipolysis in vitro. Here, after pancreatin addition, medium flow was additionally introduced. After that, the procedure was the same as for the one-step flow-through lipolysis. For comparison, a one-step flow-through experiment without pancreatin was also performed (Figure 8). During in vitro lipolysis, the pH in the reaction vessel changed comparably in the one-step (drop by 0.6 units after 10 min) and the two-step in vitro flow-through lipolysis (drop by 0.5 units after 10 min) at a tris maleate concentration of 50 mM. As expected, pH remained constant throughout the experiment without enzyme addition, and the concentration of dissolved carvedilol was highest in the procedure without pancreatin. Consequently, the carvedilol concentration in the pellet phase was very low. These results are consistent with the dissolution study in the paddle apparatus (Figure 2).

SMEDDS and its components efficiently solubilized carvedilol during one-step and two-step in vitro lipolysis. The concentration of solubilized carvedilol was similar in both methods, although it was slightly higher in the two-step method. This may be attributed to the solubilization of carvedilol in FaSSGF prior to the initiation of lipolysis in the two-step method.

### 3.5. XPRD Results

**Figure 9:**
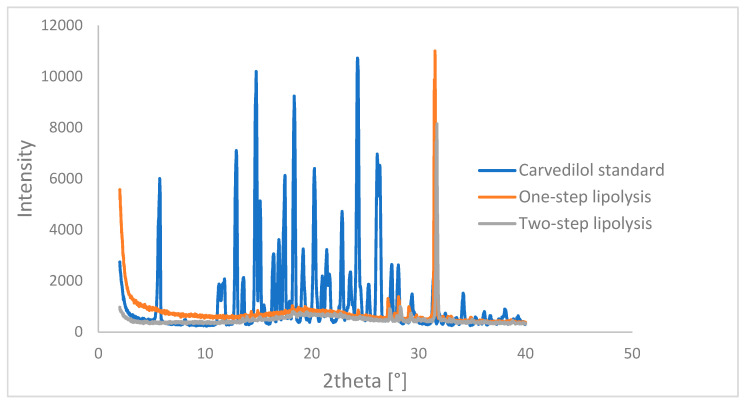
XPRD spectra of precipitated carvedilol during one-step and two-step lipolysis experiments in comparison to spectra of pure carvedilol powder.

In the manufacturing of lipid-based drug delivery systems, the carvedilol is thoroughly dissolved in SMEDDS. As the carvedilol-loaded SMEDDS passes through the gastrointestinal tract, the SMEDDS dissolves and could also be subject to digestion. This could affect the solubilization of the drug and lead to its possible precipitation, which could occur in amorphous form. To further evaluate the one-step and two-step flow-through methods, the solid state of the precipitated carvedilol was characterized using XRPD, and carvedilol was found to be amorphous (Figure 9).

## 4. Discussion

LBDDS is a promising technology for improving the absorption of poorly water-soluble drugs. However, the lack of in vitro models for drug release evaluation limits its potential for wider application [1,2,6]. Therefore, a novel in vitro release method was developed that combines the enzymatic integration of in vitro lipolysis with the mechanics of the flow-through release system. The aim is to improve the ability to evaluate drug release by increasing the number of biorelevant parameters used in the in vitro lipolysis method.

The flow-through dissolution system developed at the Faculty of Pharmacy, the University of Ljubljana, enables the biorelevant simulation of various physiological parameters of the gastrointestinal tract [33]. In the present work, it was coupled with in vitro lipolysis to develop a novel in vitro lipolysis method that also simulates the digestive flow along the gastrointestinal tract. The constant flow of the medium through the reaction vessel mimics the fluid movement along the gastrointestinal tract [34,48,49] and thus contributes to the biorelevance of in vitro lipolysis. In comparison to the in vitro lipolysis method with pH-stat, the reaction mixture is not titrated with NaOH in flow-through in vitro lipolysis, but the pH is regulated by the flow-through and the buffer capacity of the medium, which improves the physiological relevance of the developed method compared to titration with a strong base. The buffer capacity of the medium can be adjusted to reduce the pH changes caused by lipid degradation, as previously described by Mosgaard et al. [28]. In their high-throughput in vitro lipolysis model, pH regulation was provided by a high buffering capacity of the medium alone; the reported pH changes were 0.1 pH units or less.

First, a one-step pH-stat in vitro lipolysis was performed. Higher pancreatin activities resulted in lower concentrations of solubilized and higher concentrations of precipitated carvedilol, which was expected since the lipolytic reaction is faster at higher pancreatin activities [50,51], and thus the solubilizing ability of SMEDDS decreases. Nevertheless, an increase in pancreatin activity may have an insignificant effect on the response when the formulation is fully digested [50].

The pancreatin activities chosen in our study are consistent with literature data for intestinal lipolytic activity in the fasting state, which ranges from approximately 100 to 1000 U/mL in healthy adults. However, these studies use different methods for measuring enzyme activity as well as different standard substrates [52,53,54,55], which makes a direct comparison impossible. Nevertheless, one of the studies collected duodenal fluid in a fasted state from volunteers; titration at pH 8 with tributyrin as standard substrate was performed for the evaluation of lipolytic activity [53]. These conditions were closest to our study; tributyrin was used for the determination of lipolytic activity of pancreatin powder, which was performed titrimetrically at pH 7.5. Interestingly, the lipolytic activities in this study were the lowest in the literature reviewed, with values around 250 TBU/mL, as read out from the graphical presentation [53]. Based on these results, a pancreatin activity of 250 TBU/mL, the lowest of the three activities in our study (Figure 3), was used for the in vitro lipolysis experiments.

In contrast, several in vitro lipolysis models described in the literature used higher pancreatin activities to simulate intestinal lipolytic degradation in the fasting state. As a rule, activity of around 600 USP/mL was reported [16,17,28,40,41,42,56]. Sassene et al. [50] state that this is the preferred pancreatin value as it is similar to the in vivo activity, with higher pancreatin activities having only an insignificant effect on the course of the reaction. The activity of 600 USP/mL corresponds to 1000 TBU/mL [50]. In addition, even small amounts of lipids contained in LBDDS can stimulate at least a partial postprandial response [3], which corresponds to higher lipolytic activities [57]. However, Tran et al. [41] suggest that one of the reasons for the lack of IVIVC may be an overestimation of enzymatic activity, particularly in relation to lipolytic activity in rats.

Conventional in vitro lipolysis is a pH-stat method; therefore, the buffer capacity is also an important parameter. In the developed flow-through method, the buffer capacity regulates the pH of the medium during the lipolytic reaction. Intestinal fluids have buffer capacities ranging from 2.4 to 13 mM/ΔpH, which in most cases has been experimentally determined by HCl addition [58,59,60,61,62]. Although in vitro lipolysis is titrated with NaOH, the buffer capacity results obtained with HCl were compared with the in vivo data as they were in agreement. Comparing the measured tris-maleate buffer capacities with the buffer capacities of intestinal fluids, the lower range of tris-maleate concentrations (up to 50 mM) is closest to the in vivo data. Within the tris maleate concentrations of 2–50 mM, the buffer capacities increase from 0.64 ± 0.09 to 12.05 ± 1.49 mM/ΔpH (Figure 4) and thus approximately cover the buffer capacity ranges of intestinal fluids.

Due to its inclusion in SMEDDS, carvedilol is introduced into the reaction vessel in dissolved form. When SMEDDS is dispersed in the high pH buffer, carvedilol begins to precipitate, which is more apparent in the microenvironment of the dispersed droplets at higher buffer capacities. Thus, the concentrations of dissolved carvedilol are highest at time 0 in the medium with the lowest buffer concentration. If the solubility is greatly exceeded, precipitation begins immediately, which can be clearly seen with 2 mM buffer. However, at higher buffer concentrations, the initial carvedilol concentration is slightly lower, and the solubilization effect of the SMEDDS components, which is also present, can be seen at the beginning of the dissolution profiles (Figure 5).

In vitro lipolysis experiments in media with different tris-maleate concentrations were also performed using a flow-through dissolution system (Figure 6). The pH was regulated only by the flow rate and buffer capacity of the medium; no titration was performed. As expected, pH was better regulated with higher-buffer-capacity media. However, lower buffer capacity values correspond more to physiological conditions [56,57,58,59,60]. Therefore, a trade-off between improving the method and maintaining physiological conditions is required when selecting the buffer capacity of the medium.

A similar influence of buffer capacity on drug release was therefore observed in one-step flow-through and pH-stat in vitro lipolysis. Carvedilol is present in dissolved form when introduced into the medium in the form of SMEDDS. The same processes are expected in the flow-through system as in the pH-stat system. A higher initial concentration of the dissolved drug means faster precipitation at the lowest buffer capacity and thus the lowest observed concentrations of the dissolved drug. On the other hand, the SMEDDS components contribute to the solubilization of the drug. All these processes are reflected in the dissolution profiles of the flow-through but are not clearly visible as the drug and SMEDDS components are continuously removed (Figure 6). Nevertheless, the differences in drug release between the different media were similar and even more pronounced in the flow-through in vitro lipolysis, as can be seen in the second part of the profiles obtained with the pH-stat method.

In addition, the influence of the buffer capacity of the medium on the release of carvedilol from SMEDDS was again recognized, although the pancreatin mass was adjusted for the respective medium based on the pancreatin activity, and the solubility of carvedilol was comparable in all media studied. As pH changes during the flow-through in vitro lipolysis, the lipolytic activity of pancreatin might fluctuate as enzyme activity is pH-dependent. It has been reported that the specific activity of human [5] and porcine [63] pancreatic lipase has a maximum at pH 7. At pH values below and above 7, the specific activity of pancreatic lipase is lower [5,64]. According to these data, the lipolytic activity could decrease during in vitro lipolysis in the flow-through process, especially in the 2 mM medium, leading to a lower degradation of the excipient. Subsequently, more solubilization of carvedilol and less precipitation would have been expected, as lower pancreatin activity hinders drug precipitation, as found when testing the effects of different pancreatin activities on drug release (Figure 3). In addition, lower pH could affect drug solubility, but in the case of weakly basic carvedilol, higher dissolution would have been expected at lower pH (2 mM tris-maleate), which contradicts the results obtained. Thus, the lower pH is probably not the reason for the differences in carvedilol release.

A possible explanation could also be the difference in the ionic strength of the media resulting from the change in tris-maleate concentration. While ionic strength can affect the droplet size of conventional emulsions [65,66,67], this is not usually reported for microemulsions composed of nonionic surfactants. Although the effect of the buffer concentration on lipid digestion is poorly understood, a beneficial effect of NaCl presence on the micellization of bile salts has been reported [68].

For the remainder of our study, a 50 mM medium was used as it is suitable due to its high buffering capacity and physiological relevance.

In the next step, an attempt was made to simulate the dispersion of SMEDDS in the stomach medium and the transfer into the small intestine. A two-step in vitro lipolysis was performed using the pH-stat method. SMEDDS was first dispersed in an acidic medium simulating gastric fluid, then the pH was increased, and the lipolytic reaction was initiated. The shift from the acidic environment to higher pH values, mimicking the transition between the stomach and duodenum, is also likely to be important for drugs that are poorly soluble weak bases, as their solubility is reduced by increasing the pH [69,70,71]. However, the pH change did not lead to additional precipitation of carvedilol (Figure 7), which is probably due to the strong solubilizing effect of the SMEDDS components or their degradation/digestion products.

A similar two-step method was developed by Klitgaard et al. [9], which was further enhanced by the inclusion of an acid-stable microbial lipase. In contrast to our study, differences in drug release were found between the one-step and two-step in vitro lipolysis methods, which were consistent with an in vivo study. However, it should be noted that the formulations studied were not classified as SMEDDS but as LBDDS and that the presence of a microemulsion after dispersion of the formulation was not detected. In our study, the dispersion of SMEDDS and exposure to the gastric medium in vitro did not appear to have a significant effect on the release of carvedilol following medium change; therefore, it is possible that the acidic pH of the gastric medium may affect performance depending on the formulation type.

Two-step flow-through lipolysis in vitro was established based on the two-step pH-stat procedure with the addition of medium flow, which was introduced simultaneously with the addition of pancreatin and pH increase. A direct comparison between the pH-stat and the flow-through method is difficult, as they differ in their basic principle. In the pH-stat method, titration with NaOH is used to keep the pH of the medium constant, allowing constant conditions and maximum function of pancreatin. The flow-through method, on the other hand, is closer to physiological conditions and thus more biorelevant, as it maintains the pH value within a certain interval with the help of the media flow and additionally suitable buffer capacity. In our model, the buffer capacity value was a compromise between allowing a constant pH and physiological buffer capacity values. The 50 mM tris-maleate buffer was chosen because its buffer capacity is in the upper range of physiological values reported in the literature [58,59,60,61,62] but also allowed the pH in the flow-through model to be maintained within the interval of 7–7.5, where the activity of pancreatin is still high [5,64]. In addition, the precipitation of carvedilol in the flow-through system can only be observed in the initial phase after the media change, as the medium in the working vessel is diluted by the continuous flow. It can be assumed that similar processes, i.e., exposure to different media, flow-through, and dilution, also occur in the gastrointestinal tract. As can be seen in Figure 8, during the two-step procedure, pre-dispersion of SMEDDS in an acidic medium facilitates the initial solubilization of carvedilol. However, after changing the medium, the release kinetics were comparable to the one-step process.

In addition, the precipitated carvedilol was in amorphous form during in vitro lipolysis in the flow-through process (Figure 9). This is consistent with the results of Stillhart et al., who also observed precipitation of carvedilol in amorphous form after lipolysis [72]. Since carvedilol is poorly soluble in the intestinal medium, the presence of the amorphous as opposed to the crystalline form favors the further dissolution of carvedilol.

## 5. Conclusions

A novel in vitro flow-through lipolysis model was developed for the evaluation of drug release from LBDDS, namely, SMEDDS. In contrast to the traditional pH-stat model, the pH of the simulated intestinal medium in the new method is regulated by the media flow rate and buffer capacity, which is a better representation of physiological conditions than titration with NaOH. The relationship between the release profiles of carvedilol under different conditions was comparable when using the flow-through and pH-stat methods in in vitro lipolysis; however, the differences were more pronounced when using the flow-through method.

A two-step flow-through method was also developed in which SMEDDS was dispersed in an acidic medium before increasing the pH and initiating the lipolytic reaction. The dispersion of the formulation in an acidic medium further contributes to the physiological relevance of the method. The result of in vitro lipolysis depends on the chosen test parameters; therefore, it is of utmost importance that the chosen parameters are biorelevant. The method developed in this study was optimized based on pancreatin activity, buffer capacity, media shift, and flow rate and thus the approach to pH regulation, which increased the biorelevance of in vitro lipolysis and thus also possibly the predictability of in vivo performance after oral administration.

## Figures and Tables

**Table 1:** Solubility of carvedilol in the digestion media pH 7.5 with different tris-maleate concentrations and pH of the digestion media after the solubility study.

Tris-Maleate Concentration (mM)	Carvedilol Solubility at 37 °C (mg/L)	pH
2	18.1 ± 0.9	7.53 ± 0.06
50	15.8 ± 1.7	7.52 ± 0.04
200	15.3 ± 1.0	7.48 ± 0.04

Results are expressed as mean ± SD; n = 4.

## Data Availability

The original contributions presented in the study are included in the article. Further inquiries can be directed at the corresponding author.

## Abbreviations

The following abbreviations are used in this manuscript:

SMEDDS	Self-microemulsifying drug delivery system.
API	Active pharmaceutical ingredient.
LBDDS	Lipid-based drug delivery system.
rDGL	Recombinant dog gastric lipase.
TIM	TNO gastrointestinal model.
ESIN	Engineered stomach and small intestine.
SIMGI	Simulator of the gastrointestinal tract.
IVIVC	In vitro in vivo correlation.
NaDC	Sodium deoxycholate.
PC	L-α-phosphatidylcholine.
USP	United States Pharmacopoeia.
BBBA	4-bromophenylboronic acid.
FaSSIF	Fasted State Simulated Intestinal Fluid.
FeSSIF	Fed State Simulated Intestinal Fluid.
FaSSGF	Fasted State Simulated Gastric Fluid.
SGFsp	Simulated gastric fluid.
SIFsp	Simulated intestinal fluid.
HPLC	High-performance liquid chromatography.
PDI	Polydispersity index.
TBU	Tributyrin unit.
XPRD	X-ray powder diffraction.
